# Effectiveness of a serological tool to predict malaria transmission intensity in an elimination setting

**DOI:** 10.1186/s12879-016-2164-0

**Published:** 2017-01-09

**Authors:** Rajika Lasanthi Dewasurendra, Janaka Nandana Dias, Nuno Sepulveda, Geethika Sharmini Abayaweera Gunawardena, Naduviladath Chandrasekharan, Chris Drakeley, Nadira Dharshani Karunaweera

**Affiliations:** 1 Department of Parasitology, Faculty of Medicine, University of Colombo, Kynsey Road, Colombo, 8 Sri Lanka; 2 MOH Holdings Pte Ltd. 1, Maritime Square, 11-25, Harbour-Front Centre, Singapore, Singapore 099253; 3 Faculty of Infectious and Tropical Diseases, London School of Hygiene and Tropical Medicine, Keppel Street, London, WC1E 7HT UK; 4 Centre of Statistics and Applications, University of Lisbon, Campo Grande, 1749-16 Lisbon, Portugal; 5 Department of Chemistry, Faculty of Science, University of Colombo, Colombo, 03 Sri Lanka

**Keywords:** Sri Lanka, Anti-malarial antibodies, ELISA, Reversible catalytic model, Sero-positivity

## Abstract

**Background:**

Sri Lanka achieved the WHO certificate as a malaria free country in September 2016, thus monitoring of malaria transmission using sensitive and effective tools is an important need. Use of age-specific antibody prevalence as a serological tool to predict transmission intensity is proven to be a cost effective and reliable method under elimination settings. This paper discusses the correlation of four anti-malarial antibodies against vivax and falciparum malaria with the declining transmission intensities in two previously high malaria endemic districts i.e. Kurunegala and Moneragala of Sri Lanka.

**Methods:**

Sera was collected from 1,186 individuals from the two districts and were subjected to standard ELISA together with control sera from non-immune individuals to obtain Optical Density (OD) values for four anti-malarial antibodies i.e. anti-MSP1 and anti-AMA1 for both *Plasmodium vivax* and *Plasmodium falciparum*. The sero-positive samples were determined as mean OD + 3SD of the negative controls. The sero-prevalence was analyzed against the demographic characteristics of the population. A simple reversible catalytic model was fitted into sero-prevalence data to predict the sero-conversion and sero-reversion rates.

**Results:**

Over 60% of the population was sero-positive for one or more antibodies except young children (<10 years). The sero-prevalence was zero in young children and very low in young adults when compared to the older age groups. The model developed for falciparum malaria that assumed the presence of a change in transmission was not significant in the Kurunegala district although significant reduction in transmission was observed when the model was used for *P. vivax* antibody data in that district. In Moneragala district however, all the serological markers indicated a change in transmission that has occurred approximately 15 years ago.

**Conclusions:**

Assessment of MSP1 and AMA1 anti-malarial antibodies of *P. vivax* and *P. falciparum* proved to be useful indicators in predicting transmission under elimination settings as prevailed in Sri Lanka. The sero-conversion rates for the two districts studied are shown to be very low or zero indicating the absence of active and/or hidden transmission confirming a “true” state of elimination at least, in the two study districts in Sri Lanka.

**Electronic supplementary material:**

The online version of this article (doi:10.1186/s12879-016-2164-0) contains supplementary material, which is available to authorized users.

## Background

**Fig. 1:**
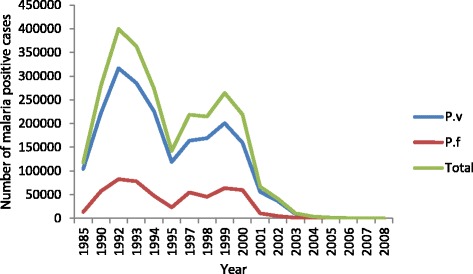
Number of malaria cases reported 1985–2008. National data on *P. vivax*, *P. falciparum* and total number of malaria cases reported from 1985 to 2008 is given

Malaria remained as a major health burden throughout history in Sri Lanka, with recurring epidemics every 3–5 years. However, countrywide malaria incidence rates started to decline during the past decade facilitated by the application of intense control programs (Fig. 1). Sri Lanka moved to the “Elimination” phase of malaria in 2013 with the last indigenous case reported in late 2012 [1, 2]. The present challenge is to maintain the status of zero transmission and prevent re-introduction of the infection since re-emergence of the disease remains a possibility. In this backdrop, accurate estimates of malaria transmission are of immense importance and relevance. Direct methods such as entomological inoculation rate (EIR) or parasite prevalence are traditionally used as indices for monitoring and evaluation of transmission [3], but utility of these tools is limited in low transmission or apparently zero transmission areas [4, 5], hence the quest for more sensitive and accurate tools.

Malaria was considered as an endemic disease in the dry zone of Sri Lanka, prior to year 2000, though its transmission was considered as low and seasonal. It peaked from December to March period during the northeast monsoons and smaller peaks occurred in concurrence with the southwest monsoon during June to October. The malaria endemic dry zone, as recognized traditionally, included mainly the northern, eastern and southeastern parts of the island with occasional epidemics in the intermediate zone (i.e. northwestern and western mountain slopes) [6]. The annual parasite incidence rate (API) reported in Sri Lanka declined markedly from 22.1 in 1999 to less than one in 2011 achieving 99.9% reduction in confirmed infections [6].

Sri Lanka has remained free of malaria transmission for the past 3 years and was certified as a state “free-of-malaria” in September 2016 by the World Health Organization (WHO) [7].

Standard molecular methods have failed so far to demonstrate the presence of submicroscopic levels of parasitaemias in previously endemic zones [8], though doubts remains as to the accuracy and sensitivity of available tools [2].

Serological markers are tools that have been recognized as good indicators of malaria transmission intensity under various malaria endemic settings with significant correlation with EIR estimates [5, 9–11]. Age-specific sero-prevalence data have also been used as evidence of reduction in malaria transmission and malaria elimination [4, 12–14]. Its robustness to detect short term variations in malaria transmission, applicability even in low transmission areas and the relatively cheaper/simpler laboratory procedures involved make this method attractive over other traditional methods [5, 10]. Furthermore, the persistence of anti-malarial antibodies for longer periods [15], enable predictions to be made even in the absence of active transmission, hence its potential utility in elimination settings. Sero-prevalence reflects cumulative exposure to malaria and therefore believed to be less affected by seasonality and/or unstable transmission [10, 16] making it more suitable for use in predicting variations in transmission.

The modeling of age-specific antibody prevalence and fitting the reversible catalytic sero-conversion model to real data has been described for *Plasmodium falciparum* (*P. falciparum*/P.f.) at varying transmission intensities [10, 17]. However, it has not been well studied in areas with *Plasmodium vivax* (*P. vivax*/P.v.) malaria transmission, though it is indeed the species that is more resistant to elimination efforts [2]. *Plasmodium vivax* had always been the more prevalent causative agent of malaria in Sri Lanka according to past records [18]. Indigenous malaria cases in the island decreased after the year 2000 with a prominent reduction of the proportion of *P. falciparum* cases. The number of falciparum malaria cases were less than five during the years 2011 and 2012 whereas the total cases were 124 and 23 respectively [2].

Anti-malarial antibodies anti-MSP1_−19_ (Merozoite Surface Protein 1_19_), anti-MSP2 (Merozoite Surface Protein 2) and anti-AMA1 (Apical Membrane Protein) have been widely used in predicting medium and long term variations of malaria transmission [4, 5, 10, 11]. However, the use of NANP (N-acetylneuraminic acid phosphatase) or CSP (Circumsporozoite Protein) appear to be limited [19].

The main objective of this study was to assess the ability to predict changes in malaria transmission by fitting age-specific anti-malarial antibody prevalence to a reversible catalytic model for both *P. vivax* and for *P. falciparum* using two anti-malarial antibodies viz. anti-MSP1 and anti-AMA1 (for both *P. vivax* and *P. falciparum*) which were proven to be sustained at high levels in Sri Lankan populations earlier [15].

## Methods

### Study area

**Fig. 2:**
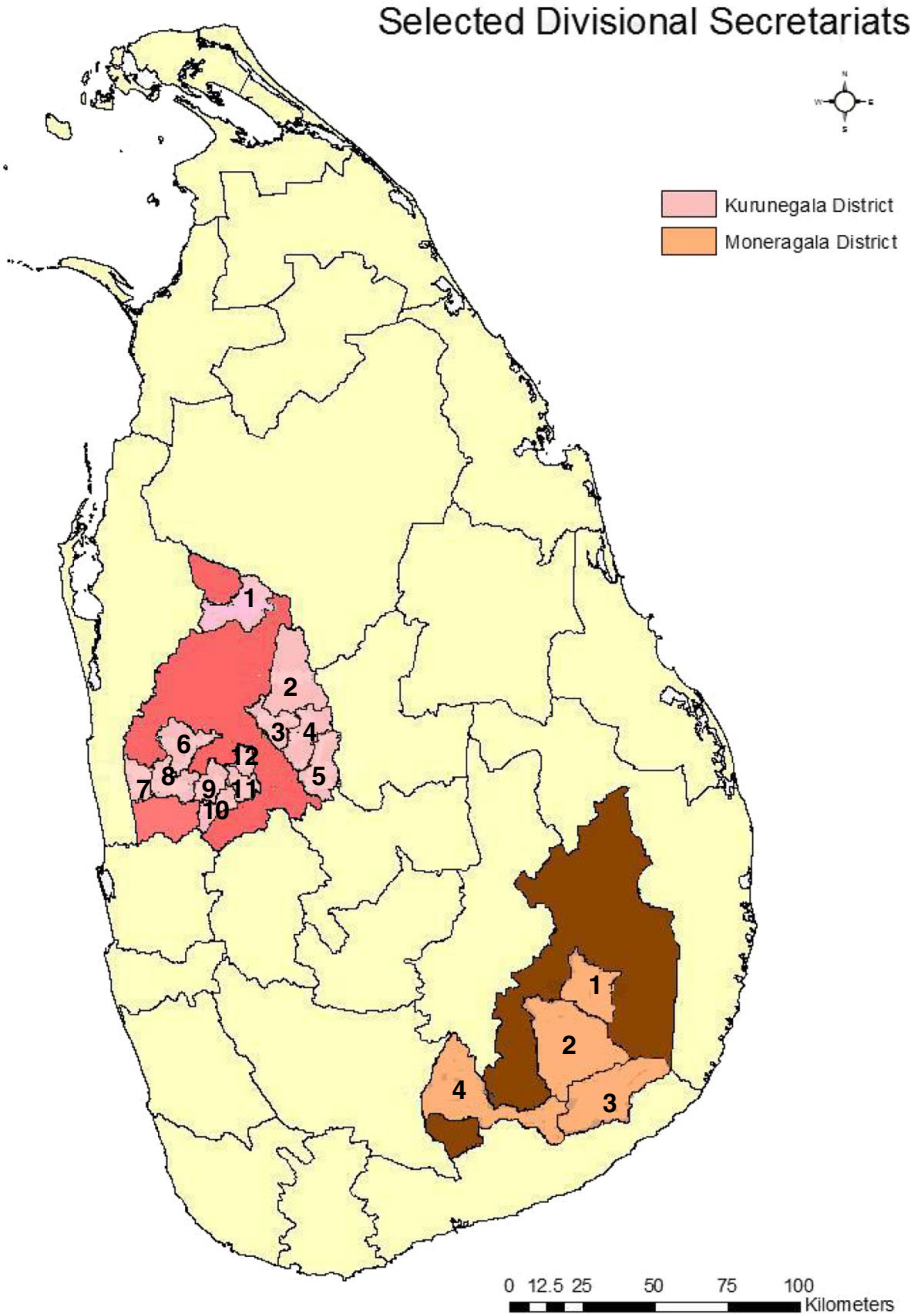
Maps of the study sites. Kurunegala district and Moneragala district. The selected DS divisions of the two districts are numbered and highlighted. Twelve DS divisions were selected from Kurunegala district (i.e. 1. Galgamuwa, 2. Polpithigama, 3. Ganewatta, 4. Ibbagamuwa, 5. Rideegama, 6. Panduwasnuwara, 7. Udabaddawa, 8. Kuliyapitiya West, 9. Kuliyapitiya East, 10. Narammala, 11. Weerambugedara and 12. Bamunukotuwa). From Moneragala district 4 DS divisions were selected (i.e. 1. Moneragala, 2. Buttala, 3. Kataragama, 4. Thanamalwila)

The present study was conducted during December 2013–August 2014 in two previously malaria endemic districts in Sri Lanka, namely Kurunegala (7.75°N, 80.25°E) and Moneragala (6.66°N, 81.33°E) located in the dry zone lowlands about 127 kilometers apart (Fig. 2). The monthly temperature of the Kurunegala district averages above 30 °C while that of Moneragala is relatively low between 25–30 °C. The mean monthly rainfall in the Kurunegala district varies from 62 to 400 mm with two peaks in April–June (150–250 mm) and in October–November (300–350 mm). Mean monthly rainfall of the Moneragala district varies from 110 – 152 mm with over 80% of rain received during the two rainy seasons i.e. October – January and March – May. Malaria transmission rates were high in both these districts until the year 2005 or so [18] when the API declined drastically (Table 1).

**Table 1:** Annual Parasitic Index (API) of Kurunegala and Moneragala districts from 2000 - 2010

Year	Annual Parasitic Index (API)	
	Kurunegala district	Moneragala district
2000	1.0 – 10	>100
2005	0.1 – 1.0	0.1 – 1.0
2010	<0.1	0.1 – 1.0

Source: Premaratna et al. (2011)

### Socio-demographic profile of the districts

Kurunegala district is considered “a more developed/urbanized area” with more municipal and urban areas compared to the Moneragala district. Furthermore, 1.9% of the population of the Kurunegala district is categorized as “urban” while the entire population of the Moneragala district is categorized under either “rural” (98.1%) or “estate” (1.9%) population, the latter being the resident labourers of areas with plantations (Table 2). By 2012 Moneragala had 21.6% of its population migrated from other districts (i.e. mainly from Badulla and Hambantota districts, which are considered as malaria endemic zones). In Kurunegala district however, the migrant population (12.1%) was from Kandy, Gampaha and Puttalam districts where malaria was not abundant (except in Puttalam). In summary the socio-demographic characteristics differ between the two districts, with the Kurunegala district having a more urbanized population with a higher population density and a considerable percentage of the community engaged in services and industries when compared to the Moneragala district, where the population is more of a rural nature engaged in agricultural activities (Table 2).

**Table 2:** Socio-demographic profile of the two districts

Socio-demographic factor	Kurunegala district	Moneragala district
Area	4,816 km^2^	5,636 km^2^
Percentage from the total population of Sri Lanka (2012)	7.9%	2.2%
Population growth factor (2001–2012)	0.97	1.21
Population density Persons/km^2^ (2012)	350	82
Urban population	1.9%	-
Rural population	97.7%	98.1%
Estate population	0.5%	1.9%
Number of housing units (2014)	2100	460
Percentage of houses with electricity	85.1%	69.4%
Percentage acquired higher education	18.5%	11.4%
Percentage not ever attended a school	3.7%	7.8%
Literacy	94%	91%
Labour force participation	57.8%	63.7%
Percentage of males in the labour force	78.1%	81.8%
Percentage of females in the labour force	41.2%	46.9%
Percentage of the labour force in agriculture	35.0%	56.9%
Percentage of the labour force in industries	26.6%	13.0%
Percentage of the labour force in government/private sector services	38.4%	30.1%
Economically active percentage population (male/female)	60.8%, 39.2%	61.8%, 38.2%

Source: Labour Force Survey 2014, Annual Report and Final Report on Population Census 2012; Department of Census and Statistics, Sri Lanka

### Sampling

All the District Secretariat (DS) divisions in the two districts (i.e. 30 from Kurunegala and 11 from Moneragala) were initially included in the sampling frame. Multi stage cluster sampling was used in a non- random basis (to identify the initial clusters) and random basis (to identify second stage clusters). Four DS divisions (out of the 11 DS divisions) from Moneragala district and 12 DS divisions (out of 30) from the Kurunegala district were selected on the basis of the presence of District/Base hospitals or Medical Officer of Health offices (Fig. 2). Second stage clusters were determined after selecting the initial clusters. Two Grama Niladhari (GN) divisions were randomly selected from each selected DS division by drawing lots (i.e. 8 and 24 GN divisions from Moneragala and Kurunegala respectively). Identification of locations was done within the secondary clusters, after obtaining the relevant area maps from the Division of Survey and Mapping of the Department of Census and Statistics, Sri Lanka [20]. Non random sampling (quota sampling) was carried out to recruit individuals for specific age groups.

### Sample size calculation

The base sample size was manually calculated (based on the district prevalence) at a prevalence of 83% (recorded sero-prevalence in Moneragala district being 83% [15]), and at 95% confidence level and with a 5% margin of error. The final sample (*n* = 500 for each district) was calculated after correcting the base sample size for differences in design, contingencies, laboratory errors etc. An approximate sample size of 500 also warrants a 50–75% relative length of the 95% confidence interval for estimating sero-conversion rate while studying Southeast Asia populations with entomological inoculation rate (EIR) equal to 0.01 [21]. Furthermore, a sample size of ~500 subjects per district seems sufficient to detect sudden drops in transmission intensity occurred at least 10 years ago under different epidemiological settings [22]. However, the selected sample size does not seem to detect abrupt changes that may have occurred in the short-term with high statistical power. It is therefore, expected that the sample sizes used in this study would be unable to detect more than two points of changes (i.e., both short-term and long-term changes) in the data.

The number of individuals to be included from each DS division was calculated based on probability proportionate to the size of the populations of each DS division.

### Ethical considerations

Ethical clearance for this study was granted by the Ethics Review Committee, Faculty of Medicine, University of Colombo, Sri Lanka.

### Sample collection

Blood samples were collected by house to house visits, from people attending communal gathering places and local health facilities. Prior approval to collect blood samples from persons attending hospitals or from the community was obtained from the relevant administrative bodies. The required numbers of samples were collected from these selected individuals after obtaining informed consent. Blood samples from children under 12 years were collected from those who attended the local hospitals and already bled for other tests. Consent for minors was obtained from their parents/guardians.

### Data collection

Every individual who was recruited for the study was given a unique identification number. All personal identifiers (e.g. name, address, and contact numbers) were entered in a data collection book. Other information i.e. age, gender, history of past malaria attacks (during their lifetime) as recalled by the individual and socio-economic data was entered to the data collection sheets prepared under each individual’s unique identification number.

### Blood collection and processing

Two milliliters (mL) of blood was drawn by a trained technician or a nurse. This was divided in to two tubes; (1 mL each) one coated with EDTA (Ethylene Di-amine Tetra-acetic Acid) and one plain tube, labeled with each individual’s identification number. The samples in EDTA tubes were stored in freezers at the end of blood collection sessions until transfer to the laboratory in Colombo, where they were stored at −70 °C for future use. Blood in the plain tube was allowed to clot for 4–5 h at 4 °C and was centrifuged at 12,000 r.p.m. (rounds per minute) to separate the serum. Serum was carefully extracted to a separate eppendorf tube labeled with the identification number and was sealed with parafilm. These serum samples were stored in the freezers until transfer to Colombo in frozen condition, in ice packs and were stored at −20 °C until further use.

### Standard ELISA to identify the sero-positive samples

A standard ELISA (Enzyme Linked Immuno-Sorbent Assay) protocol described elsewhere [15] was used with minor adjustments as follows.

Four selected malaria antigens which are described in literature [5, 10, 17] were used in this ELISA i.e. MSP1_−19_-Pf, MSP1_−19_-Pv (School of Life Science, University of Edinburgh, United Kingdom); AMA1-Pf or AMA1-Pv (Biomedical Primate Research Centre, The Netherlands). ELISA plates (96 micro-well plates/Nunclon, Germany) were coated with 50 micro liters (μL) of antigen with a concentration of 0.5 μg/mL. Plates were incubated at 4 °C overnight and were washed three times with Phosphate Buffered Saline with 0.05% Tween 20 (PBS/T). To each well 100 μL of diluted serum samples (1: 200 for MSP1_−19_ and 1: 400 for AMA1 in 2% skimmed milk in PBS/T) were added. Each ELISA plate contained 46 test samples in duplicate, a known positive control in duplicate, a known negative control and a blank well. The plates were incubated at 4 °C overnight. They were washed three times in PBS/T and 50 μL of horse radish peroxidase-conjugated rabbit anti-human IgG (Sigma-Aldrich/Cat#A8792) diluted in 1: 2000 in PBS/T was added to each well. This was incubated for 3 h at room temperature before washing three times in PBS/T. To each well 50 μL of o-phenylenediamine dihydrochloride (OPD) substrate solution (Sigma-Aldrich/Cat#P1526) (0.4 mg/mL in solvent) was added. This was left for 15 min for the colour to develop. The reaction was stopped by adding 25 μL of 2 M H_2_SO_4_ per well. Then the plates were read at 492 nm using an ELISA micro-plate reader. The Optical Density (OD) value for each well was recorded separately. Standard procedures were carried out to maintain the validity of the protocols. The same positive control was included in every ELISA plate and a chart was maintained to identify any significant deviations (+/− 2 standard deviations (SD)) of the OD value of the particular sample for normalization of the data. Plots were obtained to check cross reactivity (Additional files 1 and 2).

The baseline (cut-off values) for each antibody was determined with OD values obtained with serum samples of 16 non-exposed, non-immune apparently healthy individuals from Matara and Colombo districts (malaria non-endemic areas) and the cut-off values were determined (as mean) OD + 3SD i.e. 0.1115 for MSP1-Pf, 0.168 for MSP1-Pv, 0.0411 for AMA1-PF and 0.054 for AMA1-Pv) for positive and negative responses to each antigen according to previously used methods [10].

### Mathematical and statistical methods

The OD values for each test serum sample were entered into an Excel data sheet together with other information collected on the sample (e.g. location, age, gender, previous malaria exposure). The samples with an OD value exceeding the cut-off value for each antibody was considered as sero-positive for the relevant antibody. The total sero-prevalence, as well as gender and age specific sero-prevalence was determined for each district, DS division and GN division and was compared using Chi squared test with SPSS V15.0 statistical software. Reversible catalytic models were fitted to each sero-positivity data using a Binomial-product distribution for the sampling distribution and estimation methods based on maximum likelihood principle [5, 10, 23]. In each analysis, sero-conversion and sero-reversion rates (SCR and SRR, respectively) were estimated for each district first assuming constant transmission intensity over time (Model 1) and then assuming a change of transmission intensity (Model 2). In the latter, the past and present SCR and the time of change of transmission intensity were estimated using the profile likelihood method. Since models one and two are nested on each other, the comparison of these models was performed using the log-likelihood ratio test. A significance level of 5% was used in this test. Finally, all statistical analysis was conducted in the software package R (3.1.0); the respective scripts are available from the authors upon request.

## Results

### Characteristics of the population

A total of 1,186 individuals were recruited from the two districts (637 from Kurunegala and 549 from Moneragala). The number of females (*n* = 469) recruited from Kurunegala district was over three times higher than the male participants (*n* = 150) but the number of males (*n* = 263) was comparable to the number of females (*n* = 286) in the Moneragala district. Gender of 18 participants from the Kurunegala district has not been recorded (Table 3). The proportion of males to females recruited from the two districts was significantly different (Chi = 96.54, *p* < 0.001).

**Table 3:** Characteristics of the population

	Kurunegala district	Moneragala district	*p* value
Total Number of recruits	637	549	
	*n* (%)	*n* (%)	
Age Distribution			
1-5 years	20 (3.1%)	71 (12.9%)	*p* < 0.05
6-10 years	13 (2.0%)	65 (11.8%)	*p* < 0.05
11-20 years	56 (8.7%)	112 (20.4)%	*p* < 0.05
> 20 years	548 (86.0%)	301 (54.8)%	*p* < 0.05
Gender			
Males	150 (23.5%)	263 (47.9%)	*p* < 0.05
Females	469 (73.6%)	286 (52.1%)	
Not recorded	18 (2.8%)	0	
Previous malaria exposure			
Yes	120 (18.8%)	120 (21.9%)	*p* = 0.507
No	415 (65.1%)	373 (67.9%)	
Not recorded	102 (16%)	56 (10.2%)	

Age of the study subjects ranged between 1–84 years (mean = 43.31 year, median = 46 years) in Kurunegala and 1–85 years (mean = 26.04 years, median = 23 years) in Moneragala with mean and median ages of subjects in Kurunegala being significantly higher than in Moneragala (*t* = 15.74, df = 1184, *p* < 0.01 and Mann–Whitney U = 88597.5, *p* < 0.001). Mean age varied between 40.2–49.07 years in the DS divisions of Kurunegala, while in the DS divisions of Moneragala it ranged between 23.8–27.3 years. Study participants were divided into 4 age groups as follows: 1–5 years, 6–10 years, 11–20 years and age higher than 20 years. Although the number recruited for the fourth age group (age >20 years) was higher when compared to the other age groups in all the DS divisions in both districts, the deviation was not significant between any of the DS divisions. Numbers of individuals recruited in all age groups (except >20 years) were more in the Moneragala district, when compared to the Kurunegala district, (Table 3; Chi = 148.499, *p* < 0.001).

In the Kurunegala district 18.8% of the study population recalled (by memory) having had one or more attacks of malaria infections (either *P. vivax* or *P. falciparum*) in the past, while in the Moneragala district the percentage of a previous history of malaria was in 21.9%. Previous malaria exposure was comparable between the two districts, with relevant data not available from 102 individuals from the Kurunegala district and 56 from Moneragala district (Table 3; Chi = 0.523, *p* = 0.507).

Previous exposure was not significantly different between any of the DS divisions within Kurunegala district, but it was significantly higher in Kataragama DS division when compared to the other DS divisions in the Moneragala district (Chi = 8.133, *p* = 0.043).

The number of individuals with previous malaria exposure was significantly higher in the 11–20 years and >20 years categories in the Kurunegala district (Chi = 10.358, *p* = 0.016) when compared to the other age groups, but this pattern was not observed among the participants from the Moneragala district. Previous exposure to malaria was not significantly different between males and females in either district (for Moneragala Chi = 1.133, *p* = 0.287, for Kurunegala Chi = 7.53 *p* = 0.110).

### Sero-prevalence of the population

Sero-prevalence of each antibody was calculated for each district for each antibody. Over 60% of the population was sero-positive for one or more anti-malaria antibodies in both districts (Table 4). Higher proportion of the population was sero-positive for AMA1 antigen in both malaria species than to MSP1_−19_ antigen (Table 4). The proportion of sero-positive individuals for each of the four tested anti-malaria antibodies was significantly higher in Moneragala with the odds of being sero-positive in Moneragala being1.4 – >5 times higher when compared to Kurunegala (*p* < 0.05) (Table 4).

**Table 4:** Sero-prevalence of tested antibodies

	Sero-prevalence (%)		*p*-value	OR (CI)
	Kurunegala	Moneragala		
MSP1-Pf	25.5	64.5	<0.001	5.31 (4.22,6.69)
MSP1-Pv	32.3	45.7	<0.001	1.76 (1.42,2.19)
AMA1-Pf	54.1	61.8	0.005	1.37 (1.11,1.70)
AMA1-Pv	52.9	61.3	0.001	1.41 (1.14,1.75)
Either Pf antibody	64.9	68.3	0.206	1.16 (0.93,1.45)
Either Pv antibody	63.8	65.1	0.664	1.06 (0.85,1.32)

Sero-prevalence for each tested antibody is compared between the two districts using the chi square test. The *p*-values and the odds ratios (calculated in relation to the Moneragala district) with 95% CI are indicated

Within each district, sero-prevalence did not significantly differ between any of the DS divisions in either district. Sero – prevalence of males and females in Moneragala districts were comparable. However, the sero prevalence between sexes in the Kurunegala district showed a higher prevalence in males, the differences of which were marginally significant for AMA1-Pf (*p* = 0.031) and for any Pv (*p* = 0.044)/any Pf (*p* = 0.045) antibodies (Chi square Test).

The sero-prevalence of the subjects in age groups 1–5 years and 6–10 years were significantly lower, when compared to the values of the individuals age > 11 years (11–20 year age group and > 20 year age group) for all antibodies in Kurunegala district (*p* < 0.01, Chi square test). In Moneragala district, this difference was even more prominent with the sero-prevalence rates being markedly low in younger age groups up to 20 years (*p* < 0.01, Chi square test).

Over 50% with a history of previous exposure to malaria were sero-positive for AMA1 antibodies in both districts (54.2% for *P. vivax*, 55.8% for *P. falciparum* in Kurunegala district and 59.2% for *P. vivax*, 56.7% for *P. falciparum* in Moneragala district). However, the sero – prevalence rate for MSP1_−19_ antigen was relatively lower among individuals with history of previous exposure in Kurunegala district (i.e. 25.8% for *P. vivax*, 31.7% for *P. falciparum*), when compared to those in Moneragala district (53.3% for *P. vivax*, 51.7% for *P. falciparum*). Though the antibody prevalence for either vivax or falciparum antigen, was high (>55%) in individuals with history of previous exposure in both districts, the association between sero-positivity and history of malaria exposure by recall was relatively poor and was not statistically significant for either district (Chi square test/Phi-Cramer’s V statistics).

### Sero – prevalence as an indicator of malaria transmission intensity

Changes in transmission intensity were identified for the two districts, with regard to MSP1_−19_ -Pf, MSP1_−19_-Pv, AMA1-Pf and AMA1-Pv antibodies separately (Table 5). The population was sub-divided in to age groups with intervals of 20 years i.e. 1–20 years, 21–40 years, 41–60 years and 61–80 years. The data was tested using two models based on assumptions (a) presence of a single force of infection (Model 1) and (b) in the presence of changes in transmission (Model 2).

**Table 5:** Sero-Conversion Rates (SCR) for the two districts for each tested antibody

*Kurunegala district*	MSP1-Pf	AMA1-Pf	MSP1-Pv	AMA1-Pv	Any Pf antigen	Any Pv antigen
Model 1						
SCR	0.0079 (0.003-0.009)	0.0785 (0.025-0.104)	0.0133 (0.054-0.197)	0.1058 (0.048-0.177)	0.0582 (0.048-0.177)	0.1114 (0.048-0.177)
SRR	0.0048 (10^−13^-1.307)	0.0608 (0.017-0.104)	0.0135 (0.003-0.124)	0.0890 (0.041-0.166)	0.0239 (0.004-5.43)	0.0576 (0.041-0.166)
Model 2						
SCR past	0.0026 (0.0006-0.03)	0.0045 (0.0025-0.0127)	0.1212 (10^−4^-0.33)	167.372 (2.66-1282.19)	0.0099 (0.002-1.282)	104.896 (2.66-128.21)
SCR present	<10^−4^ (10^−7^-1.041)	0.0597 (0.0369-0.0679)	0.0123 (10^−4^-0.04)	0.0368 (0.030-0.543)	0.0612 (0.003-5.43)	0.0340 (0.003-0.542)
SRR	0.0272 (0.006-0.06)	<10^−4^ (10^−7^-10^5^)	0.0327 (0.0-1.0)	0.1369 (10^−7^-8.111)	<10^−4^ (10^−8^-10^5)^	0.0930 (10^−87^-8.11)
Change point	12	11	30	5	13	5
*p*-value (LRT)	0.061	0.115	0.015	0.049	0.087	0.058
*Moneragala district*						
Model 1						
SCR	0.0665 (0.0131-0.188)	0.0548 (0.053-0.689)	0.0309 (10^−3^-1.36)	0.0536 (0.005-0.68)	0.0751 (0.04-0.177)	0.0653 (0.0091-0.13)
SRR	<10^−4^ (10^−7^-5.59)	<10^−4^ (10^−8^-1.88)	<10^−4^ (10^−41^-2.97)	0.0003 (10^−89^-1.88)	<10^−4^ (10^−5^-0.166)	<10^−4^ (10^−41^-2.97)
Model 2						
SCR past	0.0192 (0.0050-0.0765)	0.1749 (0.022-0.35)	0.1312 (0.02-0.4)	0.1998 (0.022-0.35)	0.2572 (0.002-1.28)	0.2935 (0.022-0.49)
SCR present	0.0011 (10^−23^-0.846)	0.0231 (0.003-0.129)	0.0022 (10^−4^-0.0026)	0.0256 (0.0035-0.129)	0.0268 (0.003-5.43)	0.0251 (10^−4^-0.269)
SRR	<10^−4^ (10^−23^-1.085)	0.0026 (10^−67^-0.149)	0.0046 (10^−7^-3.48)	0.0038 (10^−67^-1.49)	<10^−4^ (10^−87^-8.11)	0.0002 (10^−7^-3.48)
Change point	10	14	16	15	13	16
*p*-value (LRT)	<0.001	<0.001	<0.001	<0.001	<0.001	<0.001

SCR was calculated assuming constant transmission intensity (Model 1) and a change of transmission intensity at a given change point (Model 2) with 95% Confidence Intervals within brackets. Sero-reversion rates (SRR) and log-likelihood associated with each model are also indicated. *P*-values refer to comparison between models one and two using the log-likelihood ratio test (LRT)

The results obtained for the Kurunegala district using the reversible catalytic model were with several discrepancies especially with Model two that assumed the presence of a change in transmission. The results were incompatible with the observed data in contrast to the outcome of Model one which indicated a single force of infection with a sero-conversion rate (SCR) of 0.0079^-yr^ and a sero-reversion rate (SRR) of 0.0048^-yr^ for MSP1_−19_ and a SCR of 0.0785and a SRR of 0.0608 for AMA1. Although model two indicated a significant change in transmission when *P. vivax* data were considered, the probable time point of such a change failed to overlap, (with time point estimated as 30 years whenMSP1-Pv data was used and 5 years with AMA1-Pv data) (Table 5).

**Fig. 3:**
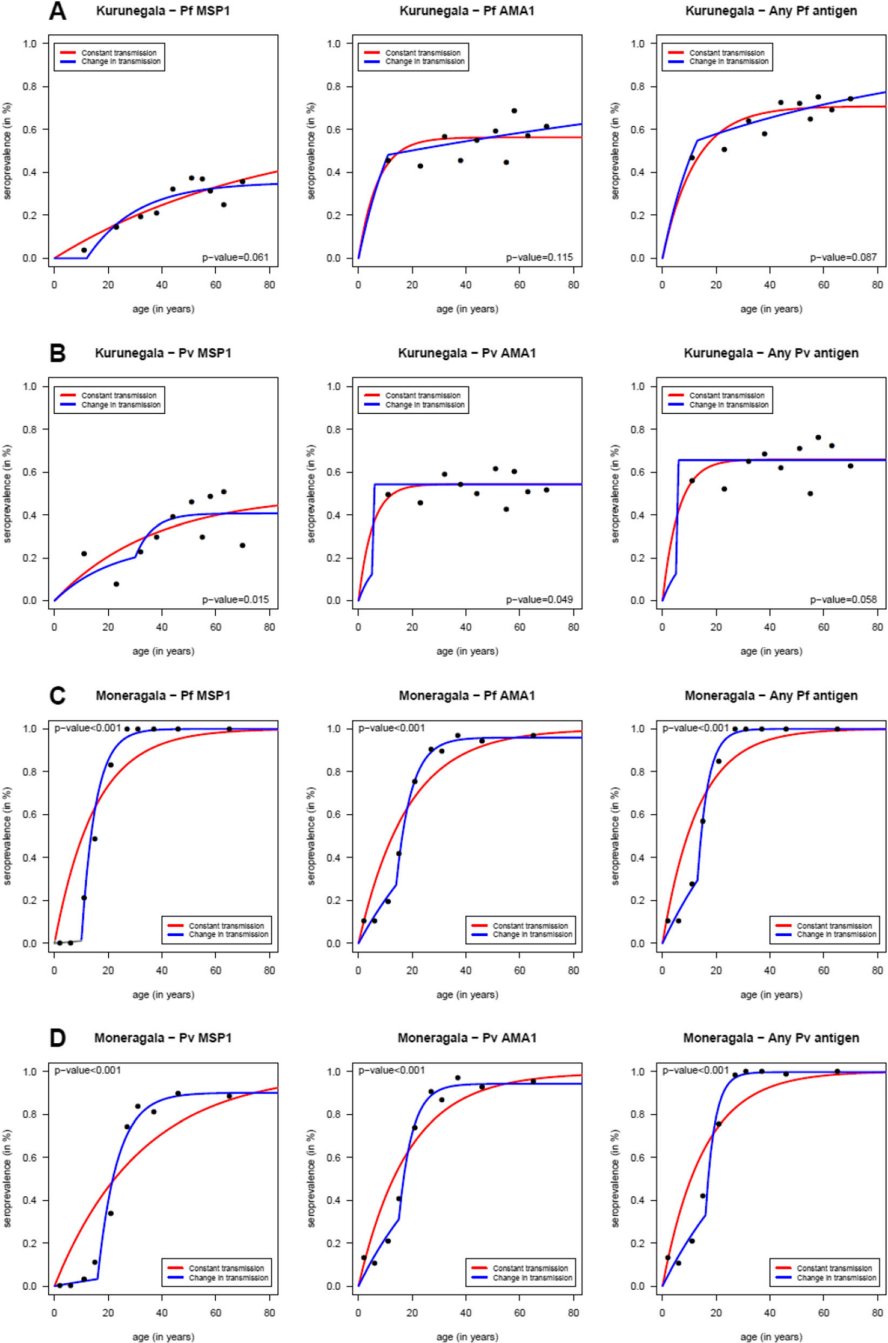
Age specific sero-prevalence curves for each tested antibody. Age specific sero-prevalence curves for Kurunegala (**a**-**b**) and Moneragala (**c**-**d**) districts for *P. vivax* and *P. falciparum* antibodies. Each graph shows the age specific sero-prevalence where the dots represent the observed sero-prevalence when the age distributions were divided into the respective 10%-centiles. Reversible catalytic models assuming a constant transmission intensity (red lines) and change in transmission intensity at a specific time (blue lines) were estimated for each tested antibody using the maximum likelihood and the profile methods, respectively. *P*-values refer to the likelihood ratio tests for comparing the two reversible catalytic models

The change of transmission for Moneragala district appears to have occurred for *P. falciparum* approximately 10–14 years and approximately 15 years ago for *P. vivax* (Fig. 3). The annual SCR decreased from 0.0192 – 0.0011 per year according to MSP1_−19_ data and from 0.1749 – 0.0231according to AMA1 data for falciparum malaria and from 0.1312 – 0.0022 per year according to MSP1_−19_ data and from 0.1998 – 0.0256 according to AMA1 data for vivax malaria (Table 5).

Sero-conversion was also calculated using the sero-positivity for any falciparum/any vivax anti-malaria antibody. This confirmed the time of change in transmission in Moneragala district, to have occurred 10–14 years and ~15 years prior to serum collection for falciparum and vivax malaria respectively (Table 5). However, data obtained for any *P. vivax*/any *P. falciparum*. antibody for Kurunegala district failed to detect a changing point in transmission.

## Discussion

Age specific sero-prevalence is an index used to assess the level of malaria endemicity in areas with both high as well as low transmission [10, 19] and as evidence of malaria elimination [4]. This study was conducted to assess the ability of using this method to identify changes in malaria transmission intensity retrospectively, in two previously endemic areas where malaria transmission is now considered virtually zero, in order to ascertain evidence for cessation of transmission.

Since the results generated should be age dependent, total random sampling could not be practiced when selecting individuals for the study. Randomization was practiced when selecting locations i.e. GN divisions from the study area, but non-random quota sampling was carried out for selecting individuals for specific age groups to ensure that each age group had an adequate number of study subjects to enable calculation of age-specific sero-prevalence in order to fit the data into a simple reversible catalytic model. However, there are several disadvantages in this method although it gives a better representation of certain groups within the population. The major drawback of quota sampling is that it is impossible to detect a potential sampling error as it is not a random sample and could lead to biased sampling. This makes it risky to project the research findings to the total population. Another limitation of quota sampling is that although it might be representative of the age, some other characteristics might not be represented proportionally in the final sample group [24].

Without active transmission it was a challenge to choose appropriate serological markers since there was little evidence for the presence of specific anti-malarial antibodies, except in the Moneragala district, where high levels of anti-malarial antibodies i.e. MSP1_−19_, MSP2, AMA1 for both *P. vivax* and *P. falciparum* and NANP (tetra amino acid repeat of the CSP protein) for *P. falciparum* were previously reported by the authors [15]. The decay rates of MSP1_−19_ and AMA1 for falciparum malaria have been shown through modeling experiments to be extremely low with antibodies having long half lives [10, 25].

AMA1 is a surface protein of merozoites which is highly immunogenic hence can be easily detected. MSP1_−19_is also described as a suitable immunological marker to assess endemicity/change in transmission levels even under low transmission conditions [10, 19].

Therefore, both MSP1_−19_ and AMA1 were selected to model transmission intensity for *P. falciparum* using age-specific sero-prevalence rates. MSP1_−19_ and AMA1 were also selected for *P. vivax* since it has been the more prevalent causative species of malaria in Sri Lanka through a major part of history and is the one that persisted longer after elimination of *P. falciparum* a few years earlier [2]. Both these antibodies appeared to be long-lived as evidenced by previous studies conducted in the Moneragala district [15].

The use of NANP as a suitable serological marker to inform malaria transmission has been debated in low transmission conditions [19]. NANP is a four amino acid repeat region in the circumsporozoite protein (CSP). Since sporozoites that enter the human body have a relatively short life span in the blood due to their rapid entry to the liver for further development or due to ingestion by macrophages, the amount of antibodies developed against CSP is believed to be very low especially in low transmission settings [19]. Therefore, CSP is considered as a less suitable and less reliable serological marker of malaria endemicity when the EIR in the area is low [26]. Thus NANP was not selected as a marker to predict transmission intensity in the Sri Lankan setting.

The number of female participants recruited from the Kurunegala district was 3 times higher than of males. In Kurunegala the blood samples were collected mainly by visiting households during the morning hours when males have already departed for work and from people attending communal gathering places e.g. village weekly fair, where the majority of attendees were females. These reasons might have influenced the results with over representation of female participants from the Kurunegala district. On the other hand the population from the Moneragala district comprised mainly farmers, *“chena”* cultivators or people who were engaged in their own small-scale businesses, and thus the male participation in the study was more or less comparable to that of females. These very reasons might also have accounted for the differences in the mean and median ages of the two districts; (mean and median ages in Kurunegala being 43 and 46 years while in Moneragala it was 26 and 23 years respectively) as well as the differences in the number of recruits in the different age groups.

Furthermore, this difference might have had an effect on the reversible catalytic model since the number of males included in the Kurunegala district was relatively low. It appears that males tended to be sero positive more than females though this difference was not statistically significant.

Past history of the disease was determined by the participants’ recall of memory. The data suggests that a history of previous exposure to malaria did not influence the sero – prevalence rates in the two districts (or between the DS divisions within each district, except in the Kataragama DS division of the Moneragala district). Kataragama, which is situated in the south of the island, is considered as a “Sacred City” with thousands of people travelling on pilgrimage each year from various parts of the country especially from the Northern areas, where malaria used to be abundant. This might have accounted for the higher transmission levels in this locality as recalled by the residents, when compared to those living in other DS divisions. These factors as well as differences in socio-economic structure in Moneragala, which is more suitable for malaria transmission could account for the higher sero-prevalence in Moneragala when compared to Kurunegala (Odds of Moneragala residents being at risk being ~ three times more than those in Kurunegala). This is further confirmed by the API indices of the two districts, with API of Moneragala being more than three times that of Kurunegala throughout the period up to year 2000 [18].

Sero-prevalence for AMA1 antigen was higher than that of MSP1_−19_ (for both *P. vivax* and *P. falciparum*). AMA1 is considered to be highly immunogenic when compared to the MSP1_−19_protein [10], which might explain the observations. Sero-prevalence among males in the Kurunegala district is significantly higher than that of females (*p* < 0.05, Chi square test), probably due to the differences in outdoor activities and attire style between the sexes. Such a pattern could not be observed in Moneragala, however, probably due to the differences in socio-economic factors and life style when compared to Kurunegala. Sero-prevalence in younger age groups i.e. <10 years was significantly low for all antibodies (*p* < 0.01, Chi square test) indicating low transmission levels in more recent years in both districts.

In the Kurunegala district however, a change in transmission was not evident when *P. falciparum* data was concerned and the modeled data indicated a single force of infection. The sero-conversion rate calculated using *P. falciparum* data was extremely low (0.0026^-yr^/0.0045^-yr^). This could happen as results of very low transmission, as described by Cook et al. [17]. As described, modeling with increasing sero-positivity with age might be inappropriate in such an instance as the increase in sero-prevalence with age could be negligible as well as a loss of sero-positivity in older individuals is also a possibility making it difficult to identify any change of point in transmission [4]. Although the model with two transmission intensities could be used for *P. vivax* data (both MSP1_−19_ and AMA1) the derived change of point in transmission through the model was 5 years prior according to AMA1 data and 30 years prior according to MSP1 data. The difference in timelines indicated by the two antibodies might be due to the differences in immunogenicity of the two antigens. AMA1 is said to be more sensitive and immunogenic of the two and this might account for the differences above. Furthermore, this might also hint at a sub-microscopic transmission that may have prevailed until 5 years prior to the sample collection. Furthermore, different transmission patterns for vivax and falciparum as evidenced by data is likely to reflect the actual difference in transmission of the two species observed over the years. However, the drop in falciparum transmission may not have occurred as suddenly as that of *P. vivax* (Fig. 1), which might be the reason that it was not evident in the sero-prevalence curves (Fig. 3).

The number of males included in the sample from Kurunegala district was significantly lower compared to females, and this might also have had an effect upon the results obtained using the reversible catalytic model, since males appear to have a higher chance of acquiring malaria and *P. falciparum* is less common compared to *P. vivax*.

In the Moneragala district the change in transmission was evident through the analysis of all tested antibodies; but pointing at slightly different time points i.e. 10–14 years ago for falciparum malaria and approximately 15 years ago for vivax malaria. Furthermore, the predicted sero-conversion rates (SCR past and SCR present) and sero-reversion rates (SRR) were different for different antibody types which were observed in both districts (Table 5). These differences might be the result of many factors, including the differences in immunogenicity (e.g. AMA1 being more immunogenic compared to MSP1_−19_), subclass of antibody, half-life of the relevant antibody and polymorphisms [19].

**Fig. 4:**
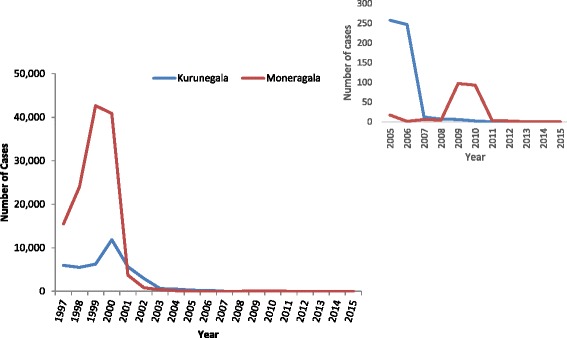
Malaria positive cases reported from the two studied districts (1997–2015). Number of total malaria cases reported from Moneragala and Kurunegala districts during 1997–2015. Number of cases reported from 2005–2015 is indicated in the inset

This study reveals a clear reduction in malaria transmission in both these districts, especially in Moneragala district where a marked decline in total district malaria cases from 40,885 to 3705 in year 2000 (Fig. 4). In Kurunegala however, according to National data there are two major drops of malaria incidence; one in year 2002 where the cases reduced from 2943 – 632 and in year 2006–2007 where the number of cases reduced from 257 – 12 cases (Fig. 4) [18]. As the sample size is not sufficient to detect more than two points change it could only be detecting the change in transmission happened around 2007 (approximately 6 years prior to sample collection) which is indicated by AMA1-Pv data (Table 5).

This reduction in transmission might be the outcome of many factors, including socio-economical changes (e.g. improved health facilities, uplifting of economical status, improved housing, better road networks), ecological (e.g. alterations of the breeding places) and interventions such as introduction of λ-Cyhalothrin in 1994, shift in IRS strategy to targeted spray in 1996–97, Roll Back Malaria initiative in 1999 and introduction of artemisinin-based combination therapy in 2007 [6].

Information considered collectively demonstrates the usefulness of serological markers to assess the endemicity under low malaria transmission conditions and its potential for use as evidence for elimination of malaria, particularly when combined with improved technology to make this tool even more sensitive and accurate.

The study used only two recombinant antigens of each *Plasmodium* species, which might be a limitation since at individual level there are likely variations in immune response to different parasite antigens. Thus there might have been individuals in the study population who did not show sero-positivity in spite of exposure to malaria. Therefore, using a higher combinations of antigens to identify sero-positive individuals might be a better approach [27].

A low number of sero-positive individuals in the younger age groups may have also limited the chance of detecting significant changes in transmission over time. A larger population of younger study subjects in the survey would have increased the chances of getting amore sero-positives within the younger age groups. Number of individuals that should be included in each age group was calculate (quota sampling) when calculating the sample size for each DS division to minimize this error. However, this had practical limitations due to difficulties in getting blood from young children in the community as malaria is not perceived as a threat in this community in recent years. Therefore, in this study blood samples were collected from younger children who attended government hospitals and private health facilities, and who were anyway bled to carry out other investigations, which limited the numbers included.

Another possible limitation to this study may have been the inadvertent inclusion of sero-positives who acquired the disease following travel to a malaria endemic country. If the travel history was not provided during the surveys, this may have led to an over estimation of the level of transmission using the serological markers. Although unlikely, with present observations, it still remains a possibility.

Cross reactivity with other parasitic antigens in the ELISA may have also influenced the results. Donahue in 2000 reported a *Toxoplasma* homolog to the AMA1 antigen of the *P. falciparum* and similarly Gaffar in 2004 documented a protein of *Babesia bovis* which was homologous to *P. falciparum* AMA1 [28, 29]. However, since the prevalence of such diseases in the Sri Lankan population is low, such a bias on observed results could be considered as negligible.

Another unlikely possibility is the cross-reactivity between MSP1_−19_/AMA1 of *P. falciparum* and *P. vivax* species. Merozoite Surface Protein 1 (MSP1) and especially Apical Membrane Antigen 1 (AMA1) are relatively conserved between both *P. vivax* and *P. falciparum* [30, 31] several studies have indicated that the cross-reactivity of these antibodies between the two *Plasmodia* species is either unlikely or very low [32, 33].

## Conclusions

Age-specific sero – prevalence curves were proven to be a useful tool in predicting malaria transmission intensity in Sri Lanka where malaria elimination has been achieved. The data suggests a low single force of transmission in Kurunegala district for falciparum malaria but hints a decline of vivax malaria starting 30 years ago. It also suggests a possibility of a sub-microscopic level transmission of *Plasmodium vivax* that may have prevailed until recently. A drastic decline in local transmission in the Moneragala district was evident, beginning approximately 10–15 years ago for both species and the current rates of sero-conversion are extremely low or zero, confirming the absence of active transmission.

## Additional files

Additional file 1: Correlation of antibodies.xlsx. Scatter-plot of OD values obtained for MSP1/AMA1 for *P. vivax* and *P. falciparum*. (XLSX 108 kb)
Additional file 2: Relationship of antibodies.xlsx. Scatter-plots of OD values obtained for antibodies of each species for the Kurunegala district and Moneragala district. (XLSX 109 kb)

## Abbreviations

°C: Centigrade
AMA1: Apical Membrane Protein 1
API: Annual Parasitic Incidence
CSP: Circumsporozoite Protein
DS division: District Secretariat division
EDTA: Ethylene Di-amine Tetra-acetic Acid
EIR: Entomological Inoculation Rate
ELISA: Enzyme Linked Immuno-Sorbent Assay
GN division: Grama Niladhari division
H_2_SO_4_: Sulfuric Acid
M: Molar
mL: Milliliters
mm: Millimeters
MSP1_−19_: Merozoite Surface Protein 1_−19_
MSP2: Merozoite Surface Protein 2
NANP: N-acetylneuraminic acid phosphatase
OD: Optical Density
OPD: O-phenylenediamine dihydrochloride
*P. falciparum*/P.f.: *Plasmodium falciparum*
*P. vivax/*P.v.: *Plasmodium vivax*
PBS/T: Phosphate Buffered Saline with 0.05% Tween 20
r.p.m.: Rounds per minute
SCR: Sero Conversion Rate
SD: Standard Deviation
SRR: Sero Reversion Rate
UK: United Kindom
WHO: World Health Organization
μg: Micro gram
μL: Micro liters
